# Research progresses on mitochondrial-targeted biomaterials for bone defect repair

**DOI:** 10.1093/rb/rbae082

**Published:** 2024-07-01

**Authors:** Shuze Wang, Jialin Liu, Linxi Zhou, Hao Xu, Dan Zhang, Xing Zhang, Qiang Wang, Qing Zhou

**Affiliations:** Liaoning Provincial Key Laboratory of Oral Diseases, School and Hospital of Stomatology, China Medical University, Shenyang 110001, China; Liaoning Provincial Key Laboratory of Oral Diseases, School and Hospital of Stomatology, China Medical University, Shenyang 110001, China; Department of Orthodontics, Shanghai Ninth People’s Hospital, Shanghai Jiao Tong University School of Medicine, Shanghai 200011, China; College of Stomatology, Shanghai Jiao Tong University, Shanghai 200011, China; National Center for Stomatology, Shanghai 200011, China; National Clinical Research Center for Oral Diseases, Shanghai 200011, China; Shanghai Key Laboratory of Stomatology, Shanghai 200011, China; Liaoning Provincial Key Laboratory of Oral Diseases, School and Hospital of Stomatology, China Medical University, Shenyang 110001, China; Liaoning Provincial Key Laboratory of Oral Diseases, School and Hospital of Stomatology, China Medical University, Shenyang 110001, China; Institute of Metal Research, Chinese Academy of Sciences, Shenyang 110016, China; School of Materials Science and Engineering, University of Science and Technology of China, Hefei 230026, China; Liaoning Provincial Key Laboratory of Oral Diseases, School and Hospital of Stomatology, China Medical University, Shenyang 110001, China; Liaoning Provincial Key Laboratory of Oral Diseases, School and Hospital of Stomatology, China Medical University, Shenyang 110001, China

**Keywords:** mitochondria-targeting, biomaterials, energy metabolism, oxidative stress, autophagy

## Abstract

In recent years, the regulation of the cell microenvironment has opened up new avenues for bone defect repair. Researchers have developed novel biomaterials to influence the behavior of osteoblasts and immune cells by regulating the microenvironment, aiming to achieve efficient bone repair. Mitochondria, as crucial organelles involved in energy conversion, biosynthesis and signal transduction, play a vital role in maintaining bone integrity. Dysfunction of mitochondria can have detrimental effects on the transformation of the immune microenvironment and the differentiation of stem cells, thereby hindering bone tissue regeneration. Consequently, targeted therapy strategies focusing on mitochondria have emerged. This approach offers a wide range of applications and reliable therapeutic effects, thereby providing a new treatment option for complex and refractory bone defect diseases. In recent studies, more biomaterials have been used to restore mitochondrial function and promote positive cell differentiation. The main directions are mitochondrial energy metabolism, mitochondrial biogenesis and mitochondrial quality control. In this review, we investigated the biomaterials used for mitochondria-targeted treatment of bone defect repair in recent years from the perspective of progress and strategies. We also summarized the micro-molecular mechanisms affected by them. Through discussions on energy metabolism, oxidative stress regulation and autophagy regulation, we emphasized the opportunities and challenges faced by mitochondria-targeted biomaterials, providing vital clues for developing a new generation of bone repair materials.

## Graphical abstract

**Figure:**
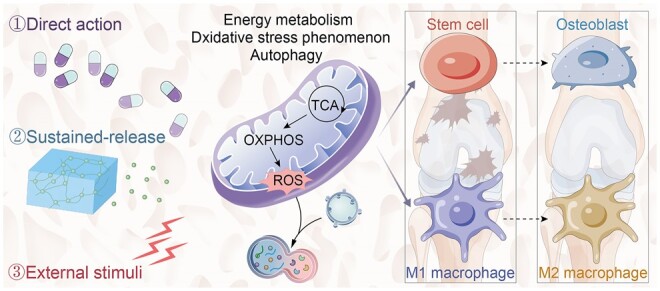

## Introduction

Repairing bone defects is crucial for maintaining normal physiological function in the human body. However, achieving ideal bone regeneration still poses significant challenges for patients with trauma, tumor resection or osteonecrosis. The process of bone repair can directly influence the stability and health of the skeleton by stimulating bone cells to promote their proliferation, differentiation and mineralization [1]. It can also indirectly promote bone reconstruction by improving the immune microenvironment, such as promoting M2 polarization of macrophages to regulate the differentiation of osteoblasts and osteoclasts [2]. However, as a traditional strategy for bone defect treatment, cell-targeted regulation faces some challenges, including difficulties in specific targeting, safety side effects, problems with biodistribution, insufficient delivery systems and the achievement of long-lasting therapeutic effects [3, 4]. At the same time, developing new strategies to regulate the M2 polarization of macrophages also faces challenges due to the lack of available safe and effective functional molecules and insufficient understanding of the dynamic temporal changes in kinase activation patterns [5]. To address these issues, mitochondria-targeted biomaterials have potential in bone defect repair [6].

Mitochondria, as pivotal organelles responsible for energy production in eukaryotic cells, play a critical role in regulating the functions of osteoblasts and macrophages concerning energy metabolism, oxidative stress and autophagy [7, 8]. Mitochondria can generate a large amount of adenosine triphosphate (ATP) to meet the metabolic needs of osteoblasts. Additionally, they can regulate the osteogenic differentiation of mesenchymal stem cells and participate in osteogenic signaling pathway transduction by coordinating oxidative stress and autophagy, thereby promoting cell generation and function [9, 10]. At the same time, mitochondria are also involved in regulating the polarization of macrophages [11]. The polarization of macrophages into either pro-inflammatory M1 or reparative M2 phenotypes is contingent upon the molecular mediators within the microenvironment [12]. M2 macrophages rely on oxidative phosphorylation (OXPHOS) to generate energy and play an important anti-inflammatory regulatory role by releasing cell factors and extracellular vesicles, effectively promoting stem cell differentiation and tissue regeneration [13, 14]. Therefore, mitochondria are of great significance in bone defect repair. It is necessary to develop biomaterials targeting mitochondria to restore the function of bone cells and the immune microenvironment, thereby promoting bone regeneration and improving treatment efficacy [15].

Compared to traditional bone-targeted regulatory methods, mitochondrial-targeted therapy has its unique advantages. These advantages are closely related to its high specificity, wide range of applications, reliable therapeutic effects and personalized treatment potential [16]. Since mitochondria are independent subcellular organelles in cells, their internal environment and metabolic pathways differ from other cellular regions. Therefore, mitochondrial-targeted technology can achieve specific regulation of osteoblasts and immune cells through local administration or local interventional surgery, thereby improving the specificity and safety of treatment [17]. At the same time, mitochondrial-targeted therapy exhibits broad applicability and can be used for various bone metabolism disorders and bone diseases, such as osteoporosis, fractures and bone defects [18]. In addition, mitochondrial-targeted therapy also shows certain potential for personalized treatment. Since the mitochondrial function status may vary among individuals, and the response to osteoblast treatment may differ, personalized mitochondrial targeting can improve the specificity and effectiveness of treatment [19]. With further research, innovative biomaterials continue to emerge, including directly acting drugs, high molecular weight materials with sustained release effects and microenvironment-responsive systems [20, 21]. These new concepts and technologies drive the continuous development of mitochondrial-targeted therapy toward precision and individualization [16]. Currently, in the field of bone repair, research on mitochondrial-targeted biomaterials mainly focuses on maintaining mitochondrial OXPHOS function, reducing reactive oxygen species (ROS) levels and maintaining mitochondrial homeostasis [22]. In animal experiments and preliminary clinical trials, promising outcomes have been demonstrated. Mitochondrial-targeted biomaterials can increase osteoblasts’ tolerance and vitality, promote bone tissue repair and regeneration and provide an important and promising research direction for bone defect repair.

**Figure 1.**
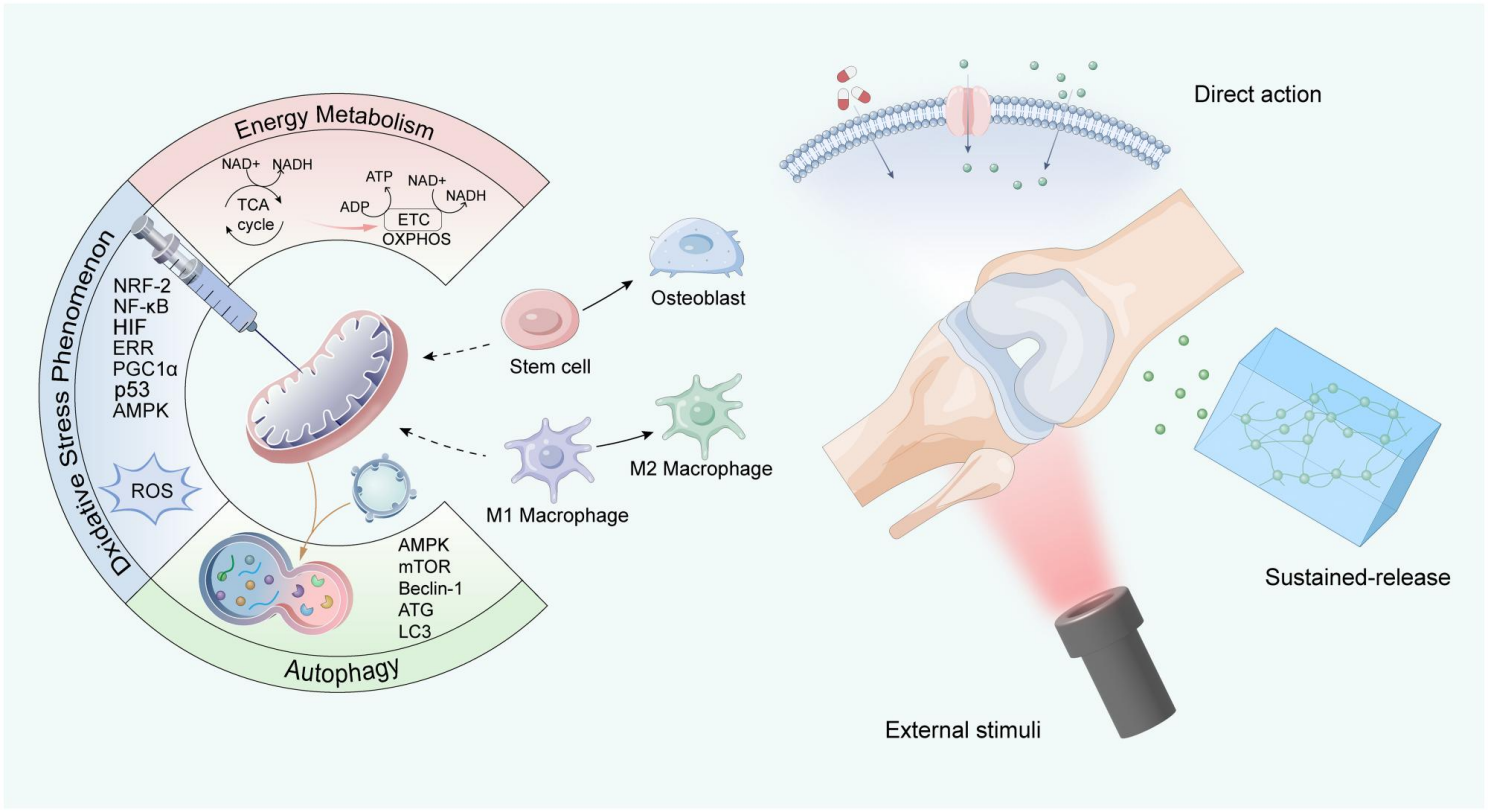
The main mode of action and mechanism of mitochondrial-targeted biomaterials for bone defect repair. Biomaterials target mitochondria through multiple mechanisms, including direct and slow-release actions, influencing mitochondrial energy metabolism, oxidative stress and autophagy. These interactions promote osteoblast differentiation and macrophage polarization, thus facilitating effective bone defect repair.

This review comprehensively discusses the important role of mitochondria in the bone healing process, specifically in osteoblasts and immune cells. We explain their potential molecular mechanisms in regulating energy metabolism, antioxidant stress and autophagy function. Recent advancements in mitochondria-targeted drug delivery systems for bone healing are also discussed, encompassing multiple drugs currently under investigation, nanoparticles designed specifically for bone tissue, and innovative materials that achieve synergistic efficacy via external stimulation. These findings are crucial not only for developing complex biotherapeutic strategies for bone defect treatments but also for identifying new targets in the prevention and management of bone metabolic disorders. Moreover, *in vivo* studies have validated the substantial potential of mitochondria-targeted nanomedicine for clinical use. This review underscores the significant application potential and scientific foundation of mitochondrial targeting biomaterials, offering vital insights and directions for future research and development in treating bone defects (Figure 1).

## Molecular mechanisms of mitochondrial-targeted therapy for bone repair

### Structure and function of mitochondria

Mitochondria, double-membraned organelles, consist of four compartments: the outer membrane (OMM), the intermembrane space (IMS), the inner membrane (IMM) and the matrix (MM) [23]. The IMM’s cristae host complex biochemical reactions, such as REDOX during OXPHOS and ATP synthesis, while the matrix facilitates the translation of mitochondrial genome proteins, the citric acid cycle and fatty acid β-oxidation [24]. Serving as cellular signaling centers, mitochondria regulate key physiological processes including cell proliferation and differentiation. They influence energy generation, signal transduction and programmed cell death, making them critical targets for organelle-specific therapies. Dysfunction in mitochondrial energy metabolism, oxidative stress, biogenesis and kinetics can lead to conditions such as osteoporosis by affecting osteoblast activity [25]. Consequently, enhancing mitochondrial function represents a viable strategy for repairing bone defects.

### Regulation mechanism of mitochondria on osteoblasts and immune microenvironment

**Figure 2.**
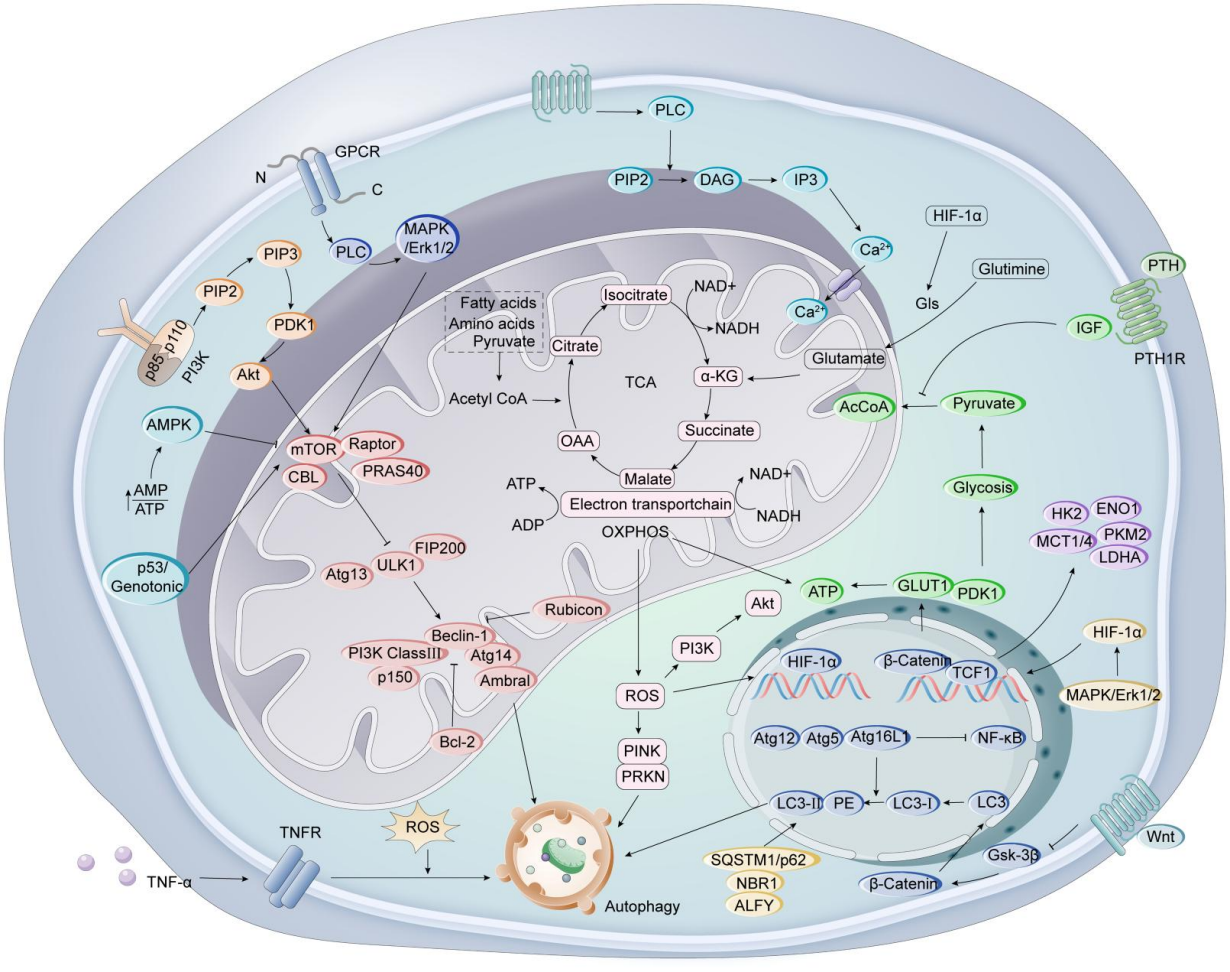
Signaling pathways associated with mitochondrial-targeted therapy. Including signaling pathways that affect mitochondrial energy metabolism, oxidative stress and autophagy.

Osteoblasts and macrophages coordinate the bone repair process to ensure the normal structure and function of the bone tissue. As the main functional cells of bone formation, osteoblasts are responsible for synthesizing, secreting and mineralizing the bone matrix, promoting continuous reconstruction of bone tissue in humans and animals [26]. Their normal proliferation, differentiation and mineralization directly affect the stability and health of the skeleton [27]. As a vital component of the innate immune system, macrophages also assume a significant function in bone repair. The timely conversion of their phenotypic activation from pro-inflammatory M1 type to reparative M2 type can affect the proliferation, differentiation, migration and apoptosis of mesenchymal stem cells while maintaining the function of osteoblasts and promoting intramembranous and endochondral ossification [28]. Mitochondria, as key organelles regulating the functions of macrophages and osteoblasts, maintain cell energy supply and signal transduction through energy metabolism, oxidative stress regulation and autophagy regulation, thereby accelerating the process of bone repair [10]. Abnormalities in these processes can lead to decreased osteogenic ability of osteoblasts, resulting in bone loss and increased fragility. It can also cause a decrease in the activity and function of macrophages, affecting their normal immune response-ability and is one of the main pathogenic mechanisms of bone metabolism diseases [29]. During the bone repair process, mitochondrial dysfunction and dysregulation in macrophages and osteoblasts can also lead to problems such as inflammation, cell apoptosis and cell dysfunction, thereby affecting the effectiveness of bone repair [30]. There are similarities in the mitochondrial targeting approaches for both macrophages and osteoblasts. Researchers focus on aspects such as mitochondrial energy metabolism, biogenesis regulation and autophagy regulation, using specific drugs or biomaterials to regulate the function and activity of mitochondria [17]. The objective is to enhance the efficiency of bone repair through targeted modulation of mitochondrial function, regulation of macrophage polarization and promotion of osteoblast proliferation and differentiation. For example, using mitochondria-specific drugs or gene regulation techniques, it is possible to regulate key factors such as mitochondrial energy metabolism products, mitochondrial respiratory chain and mitochondrial membrane potential, thereby improving cell function and inhibiting inflammation [31]. In addition, developing biomaterials based on mitochondrial metabolism and its related signaling pathways also have broad prospects for improving osteogenesis [32]. The following brief introduction will deepen the understanding of the principles of mitochondrial-targeted biomaterials through these aspects (Figure 2).

#### Mitochondrial energy metabolism

Energy metabolism, also referred to as central carbon metabolism (CCM), is the key process in which nutrients such as glucose and fatty acids are converted into energy and small molecule metabolites through processes such as the tricarboxylic acid cycle (TCA), the pentose phosphate pathway (PPP) and glycolysis within the cell. These pathways maintain basic cellular activities and provide necessary substrates for synthesizing biomolecules such as amino acids, nucleic acids and fatty acids [33]. Glycolysis, an anaerobic metabolic process, mainly occurs in the cytoplasm of cells and converts glucose into lactate, producing a small amount of ATP. On the other hand, aerobic respiration mainly occurs in the mitochondria and synthesizes a large amount of ATP through the oxidation of carbohydrates, lipids and proteins [34, 35]. The interaction and balance between these two pathways are crucial for maintaining cellular function, especially in physiological and pathological states with rapidly changing energy demands. Mitochondria, as the energy factories of cells, play a central role in bone repair regulation. Firstly, cellular energy metabolism plays a significant role in osteoblasts’ differentiation and mineralization processes, particularly in synthesizing key proteins such as collagen and bone matrix [36, 37]. Bone marrow mesenchymal stem cells (BMMSCs) primarily rely on cytoplasmic glycolysis for energy in a resting state. When BMMSCs are activated and shift toward proliferation and differentiation, they primarily rely on the mitochondria’s TCA and OXPHOS processes to produce ATP [38]. This transition in energy supply affects cell survival and has a decisive impact on its function and fate. Additionally, cellular energy metabolism plays an important regulatory role in the activation and function of the immune system [39]. To maintain homeostasis in the body, immune cells require robust glycolysis to provide sufficient energy and biosynthetic precursors. Decreased energy metabolism capacity in macrophages can lead to weakened glycolysis and mitochondrial OXPHOS, which may affect the process of bone repair [40].

Mitochondrial metabolism is involved in energy production and the regulation of intracellular signaling pathways. Metabolic products generated during the TCA cycle and OXPHOS, such as acetyl-coenzyme A (acetyl-CoA), oxidized form of nicotinamide adenine dinucleotide (NAD^+^), and pyruvate, can function as signaling molecules to regulate the function of BMMSCs [41]. For example, ROS is an important signaling molecule in regulating cellular stress responses and signal transduction [42]. Acetyl-CoA, an important metabolic intermediate, promotes the acetylation and stabilization of β-catenin, thereby activating the β-catenin signaling pathway and downstream osteogenic signaling molecules such as runt-related transcription factor 2 (Runx2) and osterix (OSX) [43]. However, with aging, mitochondria may undergo functional decline, leading to changes such as DNA damage, loss of membrane potential and increased oxidative stress. These changes can result in reduced energy, impaired bone density, increased risk of fractures and hinder the process of bone repair [44]. Therefore, a deeper understanding of the energy metabolism mechanisms in osteoblasts and osteoclasts can contribute to developing new therapeutic strategies to improve or prevent bone diseases. For example, glycolysis in osteoblasts is stimulated by various bone synthesis metabolic signals, such as parathyroid hormone (PTH), Wnt proteins, insulin-like growth factors, Notch and Hedgehog signals, which regulate aerobic glycolysis and differentiation of osteoblasts through different molecular mechanisms [13, 45]. In the early stages of repair, monocyte/macrophage lineage (MΦs) cells activate glycolytic programs to rapidly produce sufficient ATP in injury-associated MΦs, executing key effector and tissue-protective functions [46]. In the later stages of repair, a switch to OXPHOS promotes tissue protection and homeostasis during the repair response [11]. Specifically, the energy metabolism status of immune cells directly affects their repair and regenerative abilities after bone fractures or other bone injuries.

#### Mitochondrial oxidative stress phenomenon

ROS, as byproducts of aerobic metabolism, play complex and dual roles in maintaining cellular signaling and biological regulation. ROS produced mainly in the mitochondrial electron transport chain include superoxide anion ($O_{2}^{•-}$), hydroxyl radical (•OH), hydrogen peroxide (H_2_O_2_), nitric oxide (NO) and hydroxyl ion (OH^−^) [47]. ROS are important cellular signaling components under physiological conditions and can regulate cell growth, differentiation, and response to environmental changes [4]. However, when ROS production exceeds the neutralizing capacity of cellular antioxidant systems, oxidative stress occurs, damaging proteins, lipids, and DNA and affecting normal cellular functions [48]. The impact of oxidative stress is particularly significant in bone tissue. Changes in the bone environment, such as inflammation, osteoporosis and fractures, can disrupt bone cell metabolism and increase ROS production. In the process of MSC osteogenic differentiation, PGC-1α is activated by upstream signaling molecules such as AMP-activated protein kinase (AMPK), sirtuin 1 (SIRT1) and sirtuin 3 (SIRT3), which in turn affects the activity of downstream signaling molecules such as nef-1-encoded protein (NEF-1) and nuclear factor erythroid 2-related factor 2 (NRF-2). It also regulates mitochondrial function and cellular metabolic state, promoting osteoblasts’ proliferation and differentiation [49].

ROS also affects the survival of macrophages. In macrophages, ROS regulates numerous signaling pathways, including protein oxidation, activation of enzyme activity and modulation of cell surface receptor function [50]. Excessive production of ROS in macrophages can lead to cell damage and exacerbation of inflammatory reactions, so cells need to maintain a proper balance to keep normal macrophage function. Excessive production of ROS can impair cellular function, while moderate levels of ROS are necessary for successful osteogenic differentiation [51]. Therefore, precise control of ROS is crucial for maintaining the health of bone tissue and is also one of the critical factors for the success of bone tissue engineering. Mitochondrial dysfunction can lead to a series of diseases [52]. For example, in patients with periodontitis, mitochondrial biogenesis is inhibited, resulting in reduced mitochondrial number, morphological defects and functional impairments, manifested as decreased ATP production and increased ROS levels. This prompts osteoblasts to secrete tumor necrosis factor-alpha (TNF-α), inducing their apoptosis [53]. In addition, this process activates the nuclear factor kappa B (NF-κB) pathway, promoting osteoclast differentiation and osteoblast apoptosis [54]. In the periodontitis environment, macrophages’ mitochondrial dysfunction also leads to their polarization towards a pro-inflammatory phenotype, exacerbating inflammation and damage to periodontal tissues. These findings highlight the importance of maintaining mitochondrial homeostasis for the function of macrophages, osteoblasts and periodontal health.

**Figure 3.**
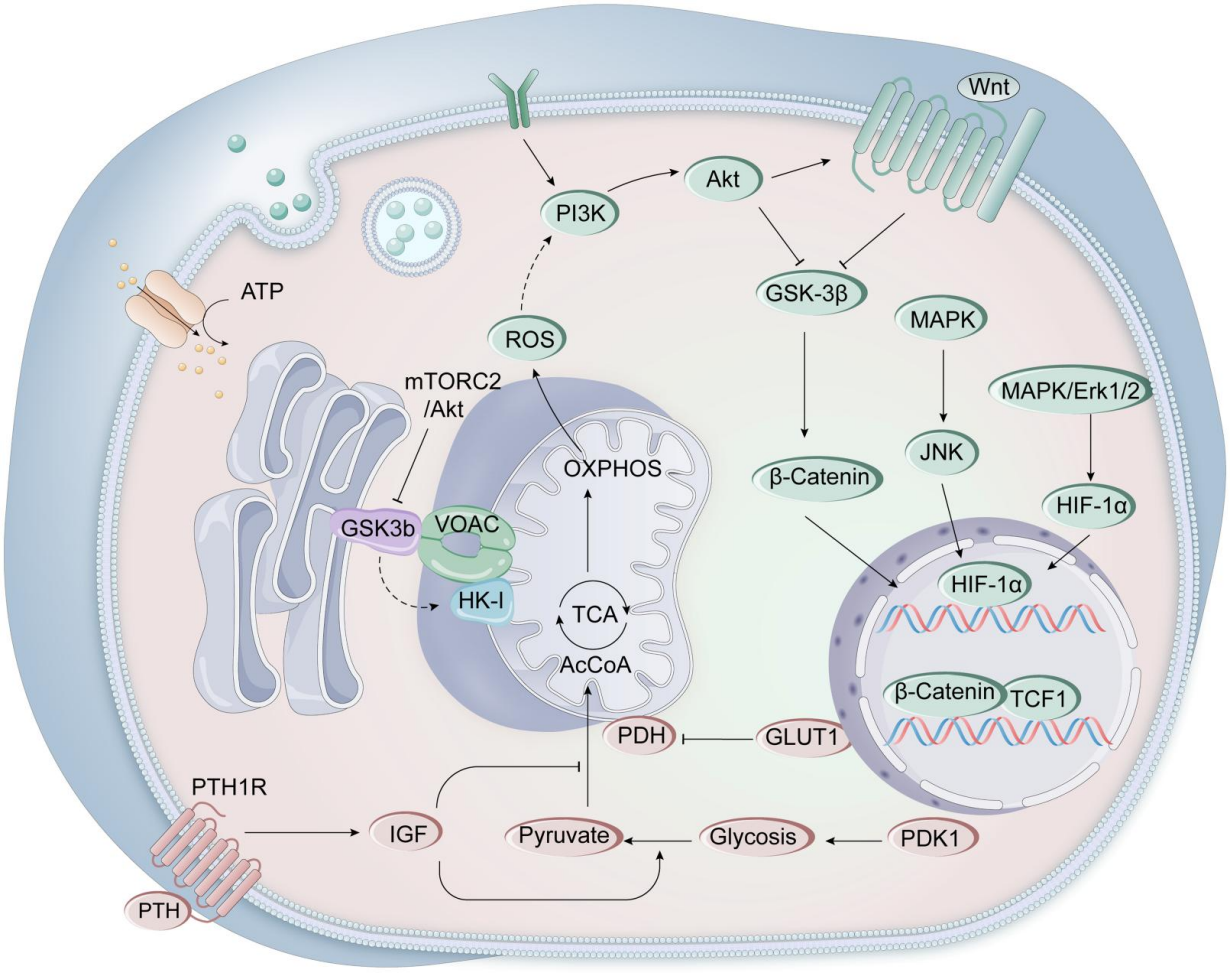
Signaling pathways associated with mitochondrial oxidative stress. Excess ROS mainly promotes β-catenin acetylation by activating phosphatidylinositol-3-kinase/protein kinase B (PI3K/Akt) pathway and improves the osteogenic differentiation potential of BMSCs.

Therefore, it is essential to have a deep understanding of the molecular mechanisms regulating ROS for bone repair. The precise control of ROS involves multiple complex molecular mechanisms. Firstly, the intracellular antioxidant enzyme system, including superoxide dismutase (SOD), glutathione peroxidase (GPx), clears excessive ROS by catalyzing reduction reactions [55]. When ROS exceeds the capacity of enzymes, cells mobilize non-enzymatic antioxidants such as glutathione, vitamin C, and E to react with ROS directly, preventing oxidative attacks on DNA, proteins and lipids [56]. In addition, there are a series of crucial redox regulatory hubs in cells, which coordinate antioxidant responses by regulating the activity of redox signaling and transcription factors. For example, NRF2 activates by oxidative modification of the inhibitory factor Kelch-like ECH-associated protein 1’s (KEAP1) Cys residue, accumulates in the nucleus and initiates the expression of antioxidant genes [57]. Other factors, such as transcription factor NF-κB, hypoxia-inducible factor (HIF) and PGC-1α, regulate the cell’s response to ROS [58]. Future research should further explore the specific mechanisms of these regulatory factors in different cell types and tissues and how they regulate the response to oxidative stress in disease states. This can provide more strategies and insights for cell protection and disease treatment (Figure 3).

#### Mitochondrial autophagy

Autophagy, a conserved mechanism for cellular clearance, contributes to the degradation and utilization of damaged or incomplete mitochondria and participates in the clearance of other organelles to support mitochondrial function and maintain cellular homeostasis [59]. The process of autophagy initiates with the formation of cup-shaped structures known as phagophores, which engulf the components to be degraded within the cell, resulting in the formation of autophagosomes. These autophagosomes then fuse with lysosomes, forming autolysosomes, where the contents are ultimately degraded, releasing energy and small molecules for cellular reuse [60]. External stimuli, such as hypoxia, oxidative stress, starvation and accumulation of unfolded proteins, can induce mitochondrial autophagy [61, 62]. Autophagy plays an important role in osteogenic differentiation. MSCs use autophagy to remove aging or damaged organelles and proteins during bone regeneration, thereby maintaining their stemness and differentiation ability [63, 64]. Osteoclasts use autophagy to clear aging cells and bone matrix in the skeleton, providing necessary conditions for bone remodeling [65]. In addition, autophagy is involved in the regulation of macrophage polarization. Research has shown that pro-inflammatory polarization of macrophages requires PTEN-induced putative kinase 1 (PINK1)-mediated mitochondrial autophagy to trigger and maintain glycolysis [66]. For example, the accumulation of acetate induced by inhibiting the activity of the pyruvate dehydrogenase complex can enhance the stability of PINK1 and induce mitochondrial autophagy by recruiting parkin. It can also promote the shift of mitochondrial respiration to glycolysis and the polarization of M1 macrophages [67].

**Figure 4.**
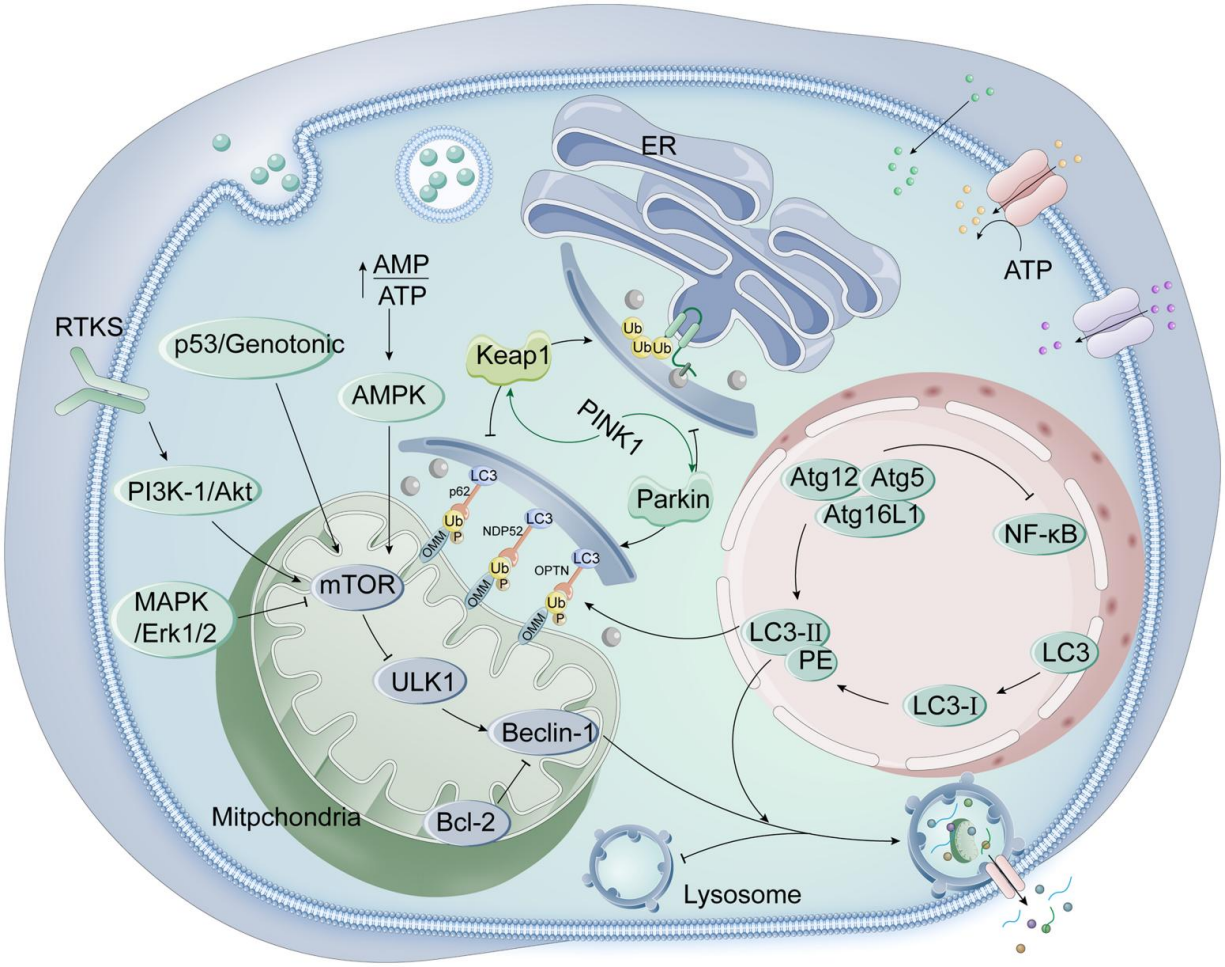
Signaling pathways associated with mitochondrial autophagy. Mitochondrial autophagy pathway is mainly divided into two types: one is the ubiquitin-dependent pathway mediated by mitochondrial kinase PINK1 (PTEN-induced putative kinase) and cytoplasmic E3 ubiquitin ligase Parkin, and the other is the non-ubiquitin dependent pathway mediated by mitochondrial autophagy receptor pathway.

Researchers have conducted related studies on the molecular mechanisms regulating autophagy. The autophagy pathway can be categorized into two primary types: ubiquitin-dependent and ubiquitin-independent pathways [68]. The former activates the PINK1/Parkin complex by reducing mitochondrial membrane potential, allowing it to interact with microtubule-associated protein 1 light chain 3 (LC3) through specific linker proteins to achieve autophagic degradation [69]. The ubiquitin-independent pathway involves mitochondrial receptors such as Bcl-2 19-kDa interacting protein 3 (BNIP3), B cell lymphoma 2-like 13 (BCL2L13) and FUN14 domain containing 1 (FUNDC1), which directly interact with LC3 to facilitate the autophagic process [70, 71]. Autophagy is also finely regulated by numerous molecules and autophagy-related proteins. The mammalian target of rapamycin (mTOR) is a core regulatory factor of autophagy, inhibiting the formation of the UNC-51-like kinase 1 (ULK1) complex, which prevents autophagy initiation, by activating signaling pathways such as AKT and MAPK [8]. Conversely, AMPK and p53 negatively regulate mTOR while promoting autophagy by activating the ULK1 and phosphatidylinositol 3-kinase (PI3K) complex [72]. Among the critical proteins in autophagy, LC3, p62 and Beclin-1 play crucial roles. LC3 is a key factor in autophagosome maturation and fusion with lysosomes, while p62 is responsible for delivering ubiquitinated proteins to autophagosomes for degradation. Beclin-1 acts as a mediator between autophagy and programmed cell death [73, 74]. These molecular mechanisms are critical in regulating cellular metabolic states and their corresponding physiological responses, especially in maintaining bone tissue health and promoting bone repair. Understanding these mechanisms is crucial for a deeper understanding of cell biology and holds important clinical significance in developing new therapeutic strategies to promote bone repair (Figure 4).

In summary, the unique role of mitochondria in the process of osteogenesis and their special physiological functions make them highly valuable and scientifically significant for targeted regulation of mitochondria. In the following content, we have summarized the targeted biomaterials for mitochondria based on different strategies such as energy metabolism, oxidative stress and autophagy regulation. The authors also consider this the most practical and feasible regulatory strategy with practical application value.

#### Others

Furthermore, mitochondrial biogenesis and dynamics play integral roles in osteoblast differentiation. Mitochondrial biogenesis, the process by which existing mitochondria produce new ones, is regulated by both mitochondrial DNA (mtDNA) and nuclear DNA (nDNA). This process involves the synthesis of mitochondrial membranes, replication of mtDNA, production of mtDNA-encoded proteins and incorporation of nDNA-encoded mitochondrial proteins [75]. The central regulator of this process is PGC-1α, an intermediate factor activated by signaling molecules such as AMPK, SIRT1 and SIRT3 [76]. Activation of PGC-1α enhances mitochondrial biogenesis, decreases ROS production, preserves mitochondrial integrity and supports the proliferation and differentiation of osteoblasts [77, 78].

Mitochondrial dynamics, the homeostatic process of continuous mitochondrial fission and fusion, is crucial for maintaining mitochondrial mass and function. This process also plays a significant role in the activities of osteoblasts and osteoclasts [79]. Alterations in mitochondrial dynamics are vital for the differentiation of mesenchymal stem cells into bone, adipose tissue and chondrocytes. The upregulation of Mfn1/2 and enhanced mitochondrial fusion facilitate osteogenic differentiation [80]. Furthermore, during osteogenesis, an increase in mitochondrial fission leads to a proliferation of small mitochondria and mitochondrial-derived vesicles (MDVs). These mitochondria and MDVs are subsequently released from osteoblasts into the extracellular matrix, which supports the maturation and differentiation of osteogenic progenitor cells, thereby promoting bone formation [81].

## Mitochondrial targeted biomaterials for bone repair

Mitochondria-targeted biomaterials offer precise treatment modalities for mitochondrial disorders by incorporating components such as carrier materials, targeting ligands, drugs and controlled release systems. The design of molecules that specifically target mitochondria is crucial for the regulation and monitoring of mitochondrial functions in the context of research and treatment of mitochondrial-related diseases. Two prevalent methods exist for mitochondrial drug delivery: direct attachment of the targeting ligand to the drug and the association of the targeting ligand with the nanocarrier [82]. The application of small molecule-based mitochondrial targeting is relatively advanced, utilizing delocalized lipophilic cations (DLCs). These DLCs, due to their lipid solubility, can traverse cellular and mitochondrial membranes, and their positive charge facilitates accumulation within the mitochondria in response to the mitochondrial membrane potential [83]. These small molecules can be directly linked to therapeutic agents, enabling precise drug delivery to the mitochondria [84]. However, challenges such as poor water solubility and potential cytotoxicity limit their broader clinical use. To address these limitations, nanoscale drug delivery systems have been developed. These nanocarriers, which include natural and synthetic polymers, liposomes, micelles, metal nanoparticles and nanoclusters, offer improved specificity for mitochondria and reduced cellular toxicity [85, 86]. Additionally, recent advances in the field have led to the development of multifunctional nanocarriers employing layered targeting strategies. These strategies allow nanocarriers to bypass biological barriers and deliver drugs effectively to mitochondria with enhanced bioavailability. Furthermore, stimulus-responsive nanocarriers enable the targeted release of therapeutics at specific sites, and synergistic therapies combine various modalities such as chemotherapy, gene therapy and phototherapy to optimize treatment outcomes [87, 88].

### Biomaterials that promote bone repair through the regulation of energy metabolism

Currently, there are still many challenges and difficulties in the effective regulation of energy metabolism. The changes in mitochondrial function are related to various factors such as environmental factors, nutritional status and exercise. Energy metabolism regulation involves a complex network of multiple molecular mechanisms and signaling pathways. The demands and regulatory mechanisms of mitochondrial energy metabolism may vary in different tissues and cell types, which increases the complexity of mitochondrial energy metabolism regulation [89, 90]. In the future, we need to investigate the mechanisms of energy metabolism further and explore more effective regulatory strategies, considering individual differences and environmental factors, to promote healthy energy metabolism.

During cellular metabolism, biomaterials such as enzymes and ions regulate cellular functions. Understanding and utilizing cellular signaling pathways and molecular biology mechanisms are essential for developing new therapeutic strategies [91]. For example, coactivator-associated arginine methyltransferase 1 (CARM1) affects cell differentiation by regulating intracellular glucose metabolism pathways [92]. CARM1 methylates the R23 site of PPP1CA, promoting osteoblast differentiation while inhibiting osteoclast differentiation. Methylation of this site affects the phosphorylation status of AKT-T450 and AMPK-T172, thereby regulating the activity of phosphofructokinase-1 and fructose-2,6-bisphosphatase 3, leading to an increase in glycolytic flux. Additionally, CARM1 regulates the expression of pyruvate dehydrogenase kinase 3, affecting the TCA cycle and OXPHOS [93]. The accumulation of magnesium ions in mitochondria also significantly regulates osteogenesis [94]. In the BMP-2-induced metabolic reprogramming, magnesium ions (Mg^2+^) may serve as a bridge connecting energy metabolism and osteogenic differentiation. After BMP-2 stimulation, there is a significant increase in the mitochondrial influx of Mg^2+^. Experimental results show that adding Mg^2+^ significantly increases cellular glycolysis and oxidative phosphorylation levels, enhancing energy metabolism and promoting osteogenesis [95].

Although many drugs have been proven to regulate cellular energy metabolism significantly, the biggest obstacle in their application is how to act and maintain drug efficacy for a long time accurately. The emergence of sustained-release regulation technology effectively solves this problem. It can better control the concentration and release time of drugs or active substances through microspheres, nanoparticles or polymers and has important application potential in bone repair processes [96].

**Figure 5.**
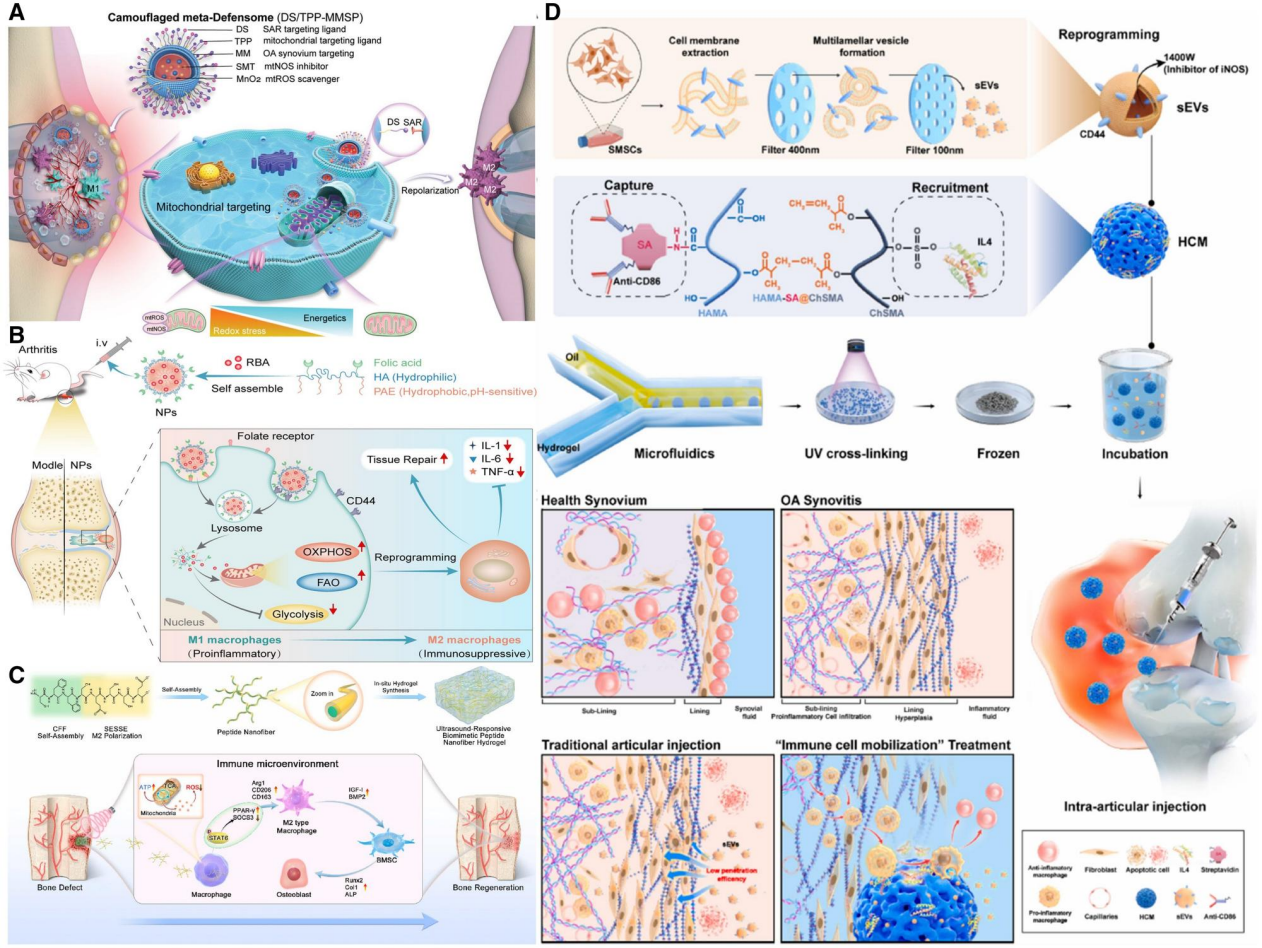
Biomaterials promote bone repair through energy metabolism regulation. (**A**) After systemic administration, DS/TPP-MMSP targeted the OA joint synovitis site. Meta-defensomes reprogramed M1 macrophages to the M2 phenotype by restoring aerobic respiration. Reproduced from Ref. [98] with permission of Wiley, © 2022. (**B**) Schematic diagram of the preparation of RBA-NPs and its therapeutic mechanism against RA. Reproduced from Ref. [101] with permission of Nature Portfolio, © 2023. (**C**) The modular self-assembly of ultra-short peptides into nanofiber hydrogels mediated macrophage M2 polarization to promote bone defect regeneration. Reproduced from Ref. [103] with permission of Elsevier, © 2015. (**D**) Schematic illustration of the preparation of immune cell mobilized hydrogel microspheres. Reproduced from Ref. [102] with permission of Elsevier, © 2023.

Scholars have proposed that glycosylated nano-hydroxyapatites (GHANPs) are a type of nanomaterial that targets the mannose receptor (MR) on the surface of macrophages. Under the stimulation of GHANPs, macrophages’ glucose uptake and conversion levels significantly increase. Cells primarily metabolize glucose through OXPHOS, and this metabolic reprogramming effectively promotes the M2 polarization of macrophages [97]. Disguised elemental defense bodies can convert M1 synovial macrophages into M2 phenotypes with an efficiency of up to 82.3%. These elementary defense bodies can clear mitochondrial ROS and inhibit nitric oxide synthase, reprogramming the mitochondrial metabolism of M1 macrophages, restoring aerobic respiration and increasing the expression of mitochondrial transcription factor A (Figure 5A) [98]. Scholars have proposed using molybdenum-containing bioactive glass-ceramic (Mo-BGC) as an immunomodulatory biomaterial, demonstrating effectiveness in periodontal tissue regeneration in animal experiments. Mo-BGC can induce M2 polarization of macrophages in the defect area and inhibit the production of multinucleated foreign body giant cells. *In vitro* experiments have shown that Mo ions released by Mo-BGC can target macrophage mitochondria and regulate M2 polarization by altering their metabolic state from glycolysis to the TCA cycle and OXPHOS [99]. The biomimetic morphology prepared on a polyether ketone (PEKK) scaffold using femtosecond laser etching and sulfonation techniques can simulate the extracellular matrix structure of liver cells, activate the MET signal of macrophages and induce immune regulation for regeneration [100]. This biomimetic morphology causes arginase 2 (ARG2) to retrotranslocate from the mitochondria to the cytoplasm, thereby increasing arginine and mitochondrial respiration in macrophages, reprogramming energy metabolism and arginine metabolism. A pH-responsive dual-target drug delivery system hitchhiking RBA (RBA-NPs), which targets CD44 and folate receptors, can effectively deliver drugs to inflammatory sites in a rat rheumatoid arthritis (RA) model. RBA-NPs also reduce glycolysis levels by blocking the ERK/HIF-1α/GLUT1 pathway, thereby promoting M1–M2 phenotype switching (Figure 5B) [101]. A microfluidic method was employed to co-blend hyaluronic acid methacrylate grafted with streptavidin and chondroitin sulfate methacrylate to prepare composite hydrogel microspheres (HCM). Through sEVs, HCM modulates the mitochondrial energy metabolism of pro-inflammatory macrophages, transforming them into anti-inflammatory macrophages in conjunction with IL4 (Figure 5D) [102]. Ultra-short peptide-formed nanofibers are loaded into alginate-calcium hydrogel triggered by ultrasound to synthesize UPN@hydrogel, which is used to fill bone defect spaces. These nanofibers can reactively be released from the hydrogel after ultrasound stimulation, reducing the production of pro-inflammatory succinate in macrophages and thereby reducing the production of inflammatory metabolic products. This material also regulates macrophage polarization to the M2 phenotype by activating mitochondrial function. In this process, macrophages’ TCA cycle and glycolysis metabolism are activated, providing the cells with additional energy and metabolic intermediates. Additionally, it is involved in regulating antioxidant stress response and cell death (Figure 5C) [103]. OI-modified DBM scaffolds (OI/CS/DBM scaffolds) regulate inflammation by metabolites, thereby regulating macrophage polarization and improving the inflammatory microenvironment of bone defects [104]. Fib-Xlink-CAD hydrogels promote osteogenic differentiation of stem cells through mechanical signal transduction-energy metabolism coupling [105]. In addition, biomaterials such as IFN-γ-microvesicles-hydrogel (Hydrogel@iMVs) and implantable polyelectrolyte hydrogel scaffolds based on DMAPS and GelMA can also enhance osteogenic differentiation by recruiting BMSCs and significantly regulating metabolic reactions [106, 107]. These studies demonstrate the combination of precise regulation of biomaterials and cellular signaling pathways, which can effectively regulate cellular behavior and metabolic state at the molecular level, providing a solid foundation for research in regenerative medicine.

Nanovesicular units (NTUs) derived from plant sources have been developed. Through chondrocyte membrane camouflage, NTUs can rapidly enter human *ex vivo* bone cells through membrane fusion, achieving cross-species applications. These NTUs have shown the ability to improve ATP and NADPH levels in degenerated joint bone cells in *in vitro* experiments, achieved by enhancing mitochondrial function and reshaping cellular energy metabolism. Specifically, NTUs increase ATP production by improving the efficiency of OXPHOS in mitochondria. In addition, NTUs regulate the activity of NADPH oxidase, further controlling the production of ROS [108]. Furthermore, it has been demonstrated that piezoelectric BaTiO_3_/Ti6Al4V scaffolds significantly promote M2 polarization of macrophages and immunomodulatory osteogenesis *in vitro*. This process involves inhibiting the MAPK/JNK signaling pathway and the activation of OXPHOS and ATP synthesis in macrophages [109]. This indicates that by altering the mechanical properties of the cellular microenvironment, the intracellular signaling pathways can be directly influenced, thereby affecting cellular fate. Moreover, these strategies help improve therapeutic efficacy and reduce unnecessary side effects, providing essential guidance for future biomedical research and clinical applications.

Biomaterials that target mitochondria to regulate energy metabolism and enhance osteogenesis typically function through both direct interactions and sustained release mechanisms. To prevent premature drug release during transport, nanocarriers are frequently engineered with REDOX, optical, magnetic and pH-responsive characteristics. These modifications help minimize damage to healthy tissues or organs. The incorporation of stimulus-responsive designs ensures that drugs are released under specific conditions, thereby increasing treatment efficacy and safety. This strategic approach holds broad potential in the biomedical field and offers novel insights and techniques for managing bone-related disorders.

### Regulation of bone repair by suppressing oxidative stress-induced production of biomaterials

It is crucial to accurately control ROS generation and maintain the antioxidant enzyme system within cells [110]. These enzymes not only specifically eliminate specific types of ROS but also achieve timely regulation of ROS clearance through fine-tuned network interactions. For example, SOD efficiently converts superoxide radicals into H_2_O_2_, which is then rapidly converted to water and oxygen by GPx and catalase (CAT), reducing the risk of ROS-induced cell damage [4]. Studies have also found challenges in applying nanomaterials in ROS generation, such as low synthesis efficiency of certain materials, which may lead to delayed physiological signaling response and affect the cell's ability to regulate ROS balance [111]. Therefore, optimizing the design and synthesis of nanomaterials and improving their interaction efficiency with the cellular antioxidant system have become key directions of current research.

In the defense system against cellular oxidative stress, direct antioxidants react directly with ROS, such as superoxide anions, hydrogen peroxide, hydroxyl radicals and nitric oxide molecules. This effectively neutralizes their activity, reducing cell damage and alleviating oxidative stress symptoms [112]. These antioxidants include naturally occurring compounds from food sources and synthetic supplements, but due to their low bioavailability, frequent intake is required to achieve sustained protection [113]. On the other hand, bioactive molecules such as melanin can also exhibit significant antioxidant effects by increasing intracellular glutathione levels and antioxidant enzyme activity and reducing levels of lipid peroxidation markers.

In specific application research of biomaterials, specific inhibitors of macrophage phagocytosis function, such as chlorpromazine, can significantly inhibit passive phagocytosis and oxidative stress levels of macrophages, reverse M1 polarization state and improve bone repair effect [109]. Ursolic acid alleviates oxidative stress by activating the Nrf2 pathway, demonstrating its critical regulatory role in cellular immune metabolism [114]. Selenite compounds significantly reduce intracellular ROS levels by regulating mitochondrial function. Selenium-enhanced biomaterials have shown potential in promoting bone defect repair in animal models [115]. However, the uncontrolled drift of rapidly released antioxidants and drugs in the body may cause immeasurable secondary damage to the body. Therefore, biomaterials that can continuously release antioxidant molecules into target tissues or cells through special release mechanisms have broader prospects for practical applications.

**Figure 6.**
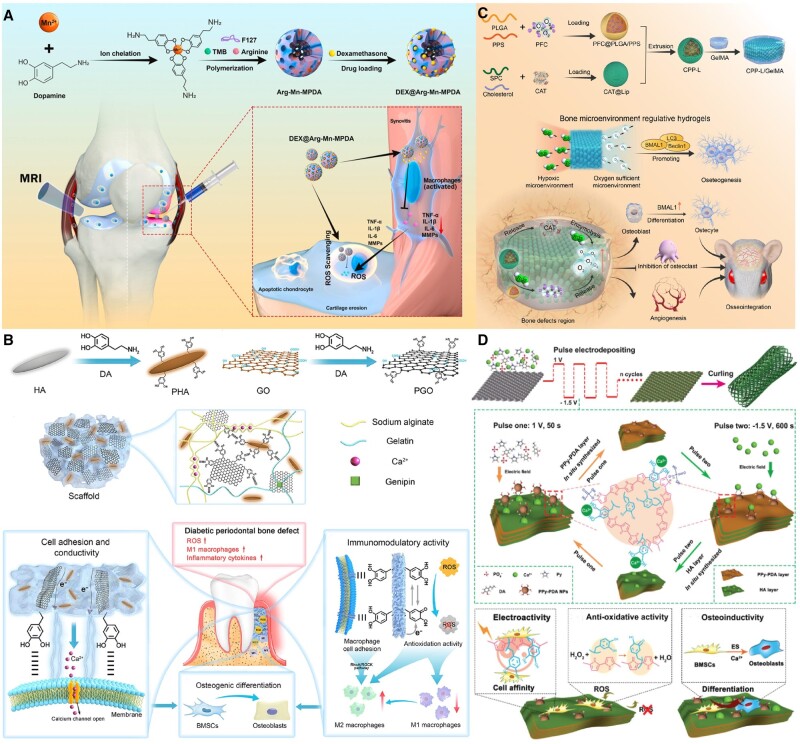
Regulation of bone repair by suppressing oxidative stress-induced production of biomaterials. (**A**) Schematic diagram of DAMM NPs for antioxidant and anti-inflammatory treatment of osteoarthritis. Reproduced from Ref. [116] with permission of Elsevier, © 2023. (**B**) Schematic synthesis of the PGO-PHA-AG scaffold with multifunctional properties that can effectively promote diabetic periodontal bone regeneration. Reproduced from Ref. [121] with permission of Elsevier, © 2022. (**C**) Schematic diagram of bone microenvironment-regulated hydrogels with ROS scavenging and prolonged oxygen-generating. Reproduced from Ref. [130] with permission of Elsevier, © 2023. (**D**) Preparation of PPy-PDA-HA-coated scaffold with electrical activity, cell affinity, antioxidant activity and osteogenic properties. Reproduced from Ref. [135] with permission of Wiley, © 2019.

For example, scholars used mesoporous polydopamine nanoparticles doped with arginine and manganese ions to release dexamethasone to treat osteoarthritis (OA) successfully. This sustained release system can regulate the interaction between macrophages and chondrocytes while effectively clearing ROS in chondrocytes (Figure 6A) [116]. In addition, arctiin can clear ROS by regulating transforming growth factor beta (TGF-β) secretion and achieve long-term and sustained drug release in a bioactive gel [117]. Similarly, scaffolds with radially oriented nSi-doped MC fibers (RA-MC/nSi) can continuously release gaseous hydrogen to selectively remove highly cytotoxic •OH and water-soluble silicic acid, promoting bone regeneration [118]. Mesoporous silica nanoparticles (MSN) doped in PDLLA-PEG-PDLLA (PPP) thermosensitive hydrogel can clear ROS generated in a high-glucose environment by continuously releasing metformin, reversing the suppressed osteogenic ability [119]. Iron-doped piezoelectric material Fe/BiOCl can combine and clear ROS generated by abnormal mitochondria through the electron energy produced by the piezoelectric effect [120]. The conductive alginate/gelatin (AG) scaffold mediated by polydopamine-coated graphene oxide (PGO) and hydroxyapatite nanoparticles (PHA) can clear ROS and anti-inflammatory activity (Figure 6B) [121]. Mn atom-substituted Co_3_O_4_ nanocrystals (Mn-Co_3_O_4_) [120], iron oxide nanoparticles (IONPs) [122], cobalt oxide supported Ir (CoO–Ir) cascade and ultrafast artificial antioxidant enzyme and Prussian blue nanozyme (PBzyme) also demonstrate significant potential in clearing ROS in the body [123, 124].

These studies indicate that biocompatible materials designed with controlled release systems have significant potential in controlling ROS levels, antioxidant stress and bone regeneration therapy [125]. However, the current design of controlled release systems still has obvious shortcomings, such as whether it can achieve local effective drug concentration and whether the system can achieve temporal regulation [126]. Microenvironment-responsive controlled release systems have been developed to inhibit the negative effects of ROS effectively. This system can dynamically adjust the release rate of antioxidants based on changes in intracellular ROS levels, thereby achieving fine control of ROS levels [127, 128]. In biomedicine, the design and application of biomaterials increasingly focus on using external physical or chemical stimuli to activate the release of antioxidants, opening up new approaches for treating ROS-related diseases. The development of smart biomaterials that can respond to specific physical or chemical signals improves the targeting and efficiency of treatment and reduces potential side effects. This makes the treatment process safer and more efficient and has important research and clinical value in enhancing the effectiveness of disease treatment [129].

Scholars have constructed a biomaterial consisting of CAT and ROS-responsive oxygen-releasing nanoparticles (PFC@PLGA/PPS) co-loaded liposomes (CPP-L) encapsulated in GelMA hydrogel. It can clear ROS and convert ROS into O_2_ responsively according to the oxygen demand in the bone defect area, thus creating a favorable microenvironment for bone regeneration (Figure 6C) [130]. In addition, the research team has successfully designed and constructed a ROS-responsive PMS/PC system. This system can regulate the wetting properties of nanopores by the oxidation of hydrophobic phenyl thioether (PhS) groups, thereby promoting the release of the antioxidant drug PC [131]. A multifunctional coating based on gallium (III)-phenol-formaldehyde network can degrade in acidic and oxidative stress microenvironments, accelerating the release of tannic acid and gallium ions, effectively reducing ROS in osteoblasts [132]. Furthermore, scholars have developed a multi-level ROS clearance system that enhances the osteogenic ability of aged bone repair by dynamically releasing drug-loaded nanomicelles to remove accumulated intracellular ROS [133]. Furthermore, hollow manganese dioxide nanoparticles (hMNP) loaded with BMP-2-derived peptides were prepared using photoacoustic imaging (PA) technology in GelMA hydrogel. This material can respond to changes in ROS in the bone microenvironment and release oxygen and BMP-2-related peptides [134]. Scholars have used layer-by-layer pulse electrodeposition technology to assemble a poly pyrrole-poly dopamine-hydroxyapatite (PPy-PDA-HA) nanofilm *in situ*. This method is new for preparing porous titanium scaffolds with electrical activity, cell affinity, persistent ROS clearance and bone-inducing properties (Figure 6D) [135]. In addition, a dual-functional coating (Ti/PDA/BP) on titanium implants, integrating two-dimensional black phosphorus nanosheets and polydopamine, can inhibit bacteria and integrate with bone implants [136]. Meanwhile, a novel ROS-responsive drug-releasing *in situ* nano hydrogel has been designed and prepared. This hydrogel utilizes ketone-thiol-based nanoliposomes that can be ruptured by ultrasound to load cartilage drugs, thereby promoting the differentiation of BMSCs towards cartilage [137]. Finally, the nano-vibration bioreactor can convert MSCs into osteoblasts through vibrational stimulation [138]. Overall, these research achievements promote the development of biomedical materials and provide new strategies and directions for treating related diseases.

In summary, the prominent application methods of biomaterials for promoting bone defect repair in response to mitochondrial oxidative stress mainly include direct antioxidants, sustained-release antioxidants, responsive sustained-release and externally triggered antioxidants. Among them, activating antioxidants released by external physical or chemical stimuli has shown great potential in precisely controlling antioxidants and treating ROS-related diseases. It is expected that in the future, these biomaterials will have broader applications in the fields of tissue engineering and regenerative medicine, providing new ideas and strategies for the treatment of oxidative stress-related diseases.

### Regulation of bone repair through intervention in mitochondrial autophagy behavior with biomaterials

As a critical intracellular degradation and recycling process, autophagy is influenced by many factors. Its regulatory mechanisms exhibit significant complexity and diversity, making it a frontier topic in biomedical research [139]. Firstly, autophagy shows diverse biological effects in different cell types, biological species, developmental stages and even under the influence of different molecular factors. This phenomenon requires precise assessment and regulation of individual differences, posing significant challenges to the clinical application of autophagy modulators. In addition, complex interactions and cross-regulatory mechanisms exist between the autophagy process and other cellular physiological processes, which place higher demands on developing precise regulatory strategies [140]. Moreover, once autophagy is initiated, if it cannot be stopped at the appropriate time, its non-specific capturing of cytoplasmic components may lead to irreversible cell damage [141]. Therefore, precise regulation of autophagy and its temporal control are key to achieving its potential therapeutic effects.

**Figure 7.**
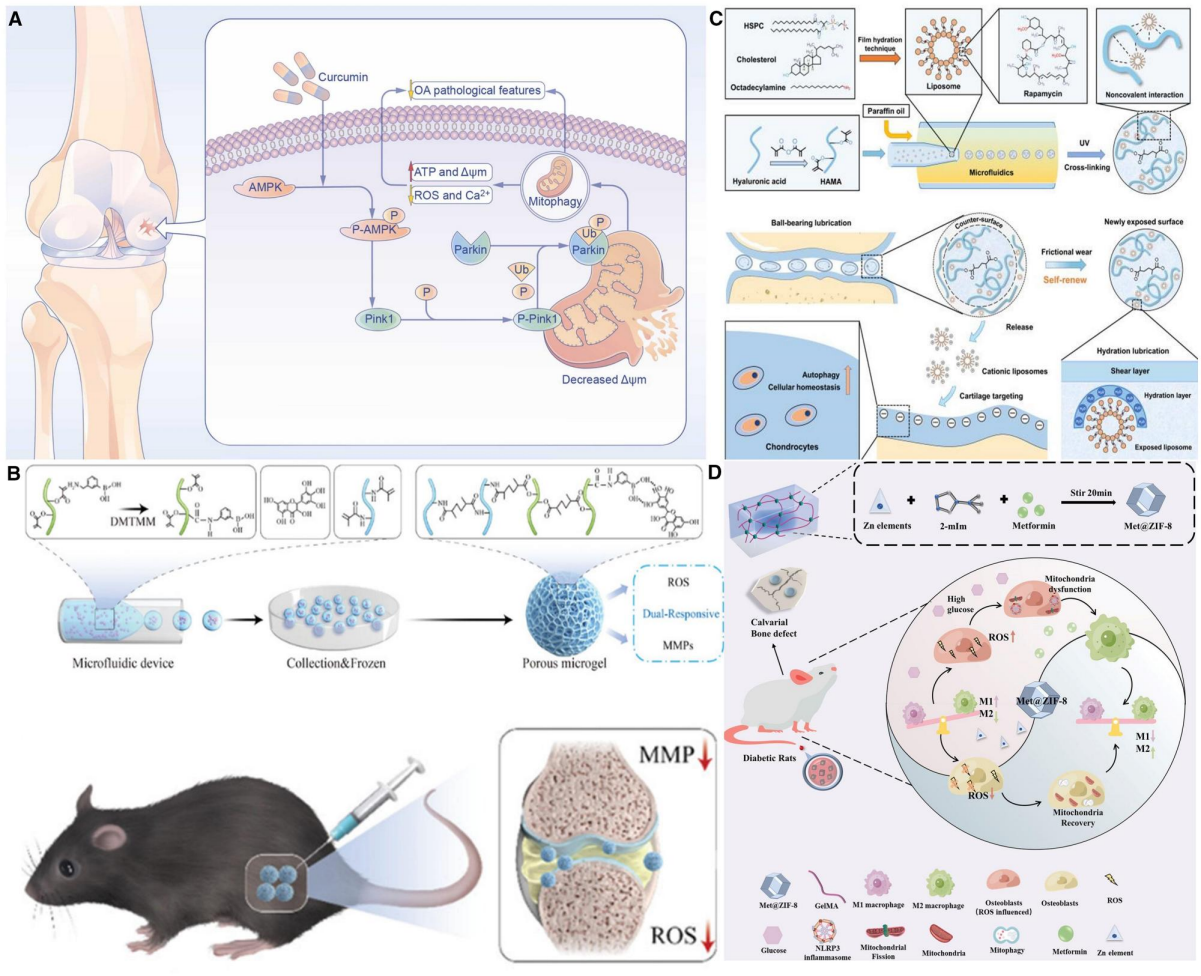
Regulation of bone repair through intervention in mitochondrial autophagy behavior with biomaterials. (**A**) Schematic illustration of the potential mechanisms by which curcumin-mediated mitophagy induction via AMPK/PINK1/Parkin pathway in the rat OA model. Reproduced from Ref. [144] with permission of Elsevier, © 2022. (**B**) Flowchart of the preparation of ROS and MMP dual-responsive hydrogel microspheres and DMY response release from microspheres in OA microenvironment. Reproduced from Ref. [151] with permission of Wiley, © 2023. (**C**) The principle and fabrication of RAPA@Lipo@HMs. Reproduced from Ref. [152] with permission of AAAS, © 2022. (**D**) Schematic illustration of how dose Met@ZIF-8 breaks the ‘vicious cycle’ in the diabetes microenvironment. Reproduced from Ref. [153] with permission of Wiley, © 2023.

This bioregulatory material mainly acts on various bone cells, promoting bone repair by increasing BMSCs osteogenic differentiation and reducing osteoblast apoptosis. Regarding direct effects, drug regulation of autophagy is an important strategy for treating various orthopedic diseases involving a series of complex molecular mechanisms. Drugs such as Vitamin D3, Simvastatin, Curcumin, Quercetin, Estrogen, Artemisinin, Genistein, Leptin (LEP), Fenofibrate (FN) and Epigallocatechin gallate (EGCG) significantly affect the autophagy process by regulating key signaling molecules and pathways in the autophagy pathway. For example, Vitamin D3 activates the CAMKK2–PRKAA signaling pathway, promoting autophagy. Simultaneously, it suppresses MTORC1 activity by elevating cytoplasmic calcium levels, crucial in preventing excessive autophagy [142]. Simvastatin, as an AMPK activator, can increase autophagy and osteogenic differentiation of BMSCs under adverse conditions [143]. Curcumin enhances autophagy and alleviates inflammatory response by activating the AMPK/PINK1/Parkin signaling pathway (Figure 7A) [144]. Quercetin improves mitochondrial and lysosomal function by activating AMPK activity and reversing oxidative stress [145]. Estrogen promotes autophagy and upregulates RAB GTPase, an activating protein precursor, to reduce osteoblast apoptosis [146]. Artemisinin regulates autophagy by inhibiting TNFSF11 expression in the IL-1β-induced OA model [147]. Genistein restores mitochondrial homeostasis and has therapeutic potential for osteoporosis caused by estrogen deficiency [148]. LEP induces autophagy to protect BMSCs from apoptosis [149]. As a PPARα agonist, FN prevents cartilage degradation by increasing autophagic flux [150]. These drugs are important in treating inflammation, osteoporosis and other orthopedic diseases by regulating the autophagy pathway and affecting autophagy initiation, execution and termination.

In terms of indirect action, intelligent drug delivery systems have shown great potential in regulating autophagy. These systems enhance therapeutic efficacy and reduce side effects by precisely controlling the release location of drugs. For example, water-based gel microspheres loaded with D-flavonoids can be directly delivered to damaged joints through intra-articular injection, thereby improving symptoms of osteoarthritis in multiple ways (Figure 7B) [151]. At the same time, self-renewing hydrate layer HMs (RAPA@Lipo@HMs) hydrogel synthesized by scholars improves joint lubrication through its smooth rolling mechanism and activates the autophagy process by releasing rapamycin (Figure 7C) [152]. In addition, zeolite imidazole salt framework-modified hydrogel loaded with metformin (Met@ZIF-8) can simultaneously release metformin and zinc elements. This synergistic effect not only affects ROS and inflammation but also maintains the stability of organelles, improves the diabetic microenvironment and enhances osteogenic performance (Figure 7D) [153].

These intelligent drug delivery systems demonstrate potential advantages in treating diseases such as osteoarthritis and complications of diabetes. Through the precise modulation of essential signaling molecules and pathways involved in autophagy, these systems offer novel strategies and theoretical frameworks for addressing autophagy-related disorders. The continued refinement and clinical implementation of these platforms will yield more efficient approaches for treating associated ailments.

Metal ions play a key role in activating autophagy and positively influencing bone homeostasis. For example, calcium ions (Ca^2+^) are involved in the autophagy signaling pathway. Under metabolic stress, the efflux of Ca^2+^ leads to an increase in cytoplasmic Ca^2+^ concentration, which can directly activate AMPK kinase or activate AMPK through Ca^2+^-dependent kinase CaMKKβ, thereby inhibiting mTOR and promoting autophagy [154]. Magnesium ions, as natural activators of calcium, can silence autophagy to accelerate osteoblast differentiation. Ion concentration plays a decisive role in the regulation of autophagy. For example, high magnesium concentrations can inhibit MAPK/ERK phosphorylation, reduce autophagic expression in chondrocytes and protect articular cartilage [155]. The regulation of autophagy also involves complex nanomaterial systems. For example, exosome-functionalized cell-free PLGA/Mg-GAMOF scaffolds, constructed with hADSCs-Exos, Mg^2+^, and gallic acid, enhance osteogenesis, anti-inflammatory and angiogenesis abilities [156]. AuNPs modified with different chiral cysteines can significantly promote osteogenic differentiation of human periodontal ligament-derived cells, possibly related to autophagy [157]. Zinc oxide nanoparticles promote autophagy by inducing autophagosome accumulation and lysosomal function. Functionally designed mitochondria-targeted silica nanoparticles inhibit the formation of dysfunctional mitochondria in MSCs, promote autophagy, and clear damaged mitochondria [158]. Sr promotes autophagy by activating AMPK and can effectively induce osteogenic differentiation of MC3T3-E1 cells [159]. These studies suggest that intelligently designed nanomaterials and metal ions can precisely regulate autophagy, providing new strategies for treating bone diseases such as osteoporosis. In the future, further optimization and clinical application of these systems will provide more effective methods for treating autophagy-related diseases.

The surface morphology of polymer materials significantly impacts cellular behavior, which is particularly crucial in designing and applying bone repair materials. For example, the surface shape, roughness, affinity, biocompatibility and chirality of materials are essential for cell adhesion and growth [160]. Rough surfaces are particularly beneficial for developing small particle cells and forming cell clusters, which are important in creating bone nodules and mineralization processes [161]. Certain surface structures, such as tubular structures, can temporarily promote autophagy during the early contact stage, potentially linked to the stretching of cell membrane structures [162]. Chiral nanomaterials augment intracellular oxidative stress and accumulation, exhibiting a notable chiral-dependent effect on inducing autophagy [157].

Nanomaterials play an important role in autophagy regulation due to their diversity and potential. These materials range from rare earths, metals and alloys to metal oxides, semiconductors, carbon-based materials and polymer nanoparticles [112]. Nanomaterials can trigger autophagy by functionalizing with bone-conductive/inductive molecules or drugs, thereby playing a role in osteogenic differentiation, angiogenesis and inhibition of bone loss. For example, materials such as nano-silica, nano-titanium, alumina, chitosan and polydopamine-templated hydroxyapatite can stimulate the autophagy process in cells through molecules or drugs attached to their surfaces [163]. These nanoparticles can deliver drugs and influence autophagy regulation in bone cells through surface morphology. Nanoparticles with specific pore sizes and surface morphologies can activate autophagy pathways, promoting metabolic activity in bone cells and tissue regeneration. By precisely designing the surface properties of nanomaterials, autophagy processes can be effectively manipulated, bringing innovative therapeutic strategies to the field of biomedical research [164].

For example, titanium and its derivative biomaterials are widely used in bone repair manufacturing due to their excellent biophysical properties. Specific titanium surface structures can significantly affect cell proliferation, migration and differentiation [165]. Scholars have studied the nanomorphology of titanium plate surfaces and explored their relationship with the Wnt/β-catenin signaling pathway, YAP protein and osteogenic differentiation. The study found that cells on the nanosurface showed changes in the expression of autophagy markers (such as LC3II/I ratio and p62 protein). Transmission electron microscopy results also showed that the autophagosomes in nanosurface cells were denser, indicating an increase in autophagy level [166]. These findings indicate that precise control of material surface morphology can effectively regulate cell behavior and autophagy processes. This regulation plays an important role in bone repair and other biomedical applications.

In conclusion, autophagy regulation involves various factors such as hormones, growth factors and metal ions, which influence osteocyte activities including growth, proliferation and differentiation. Notably, alongside methods to functionalize nanomaterials, altering material surfaces or structures to enhance cell adhesion and growth remains a pivotal research focus. For instance, modifying material properties like shape, surface roughness, affinity, biocompatibility and chirality can impact cellular autophagy, thereby facilitating bone nodule formation and mineralization. Exploring these avenues will deepen our understanding of autophagy’s role in osteocyte function and bone repair, offering innovative approaches for future therapeutic strategies and biomaterial design.

## Conclusion and viewpoint

As clinical applications of bioregenerative materials have deepened, the urgent demand within the clinic has spurred a vast market for bone repair materials. Currently, although natural allogeneic and xenogeneic bones predominantly comprise the choice of bone repair materials, synthetic alternatives have captured a significant market share in China. The products available are primarily categorized into natural bone repair materials, such as Calcined Bovine Bone, and synthetic options like YouGeSheng. These materials are typically available in granular or porous structures, suitable for the filling and repair of non-load-bearing sites. However, the diversity of existing materials remains limited, necessitating enhanced research into novel materials. Notably, there is a scarcity of solutions for filling extensive bone segments at load-bearing sites, which underscores the need for further development of materials with robust osteoinductive regeneration capacities to overcome the limitations in the timeliness and regional applicability of current materials. Mitochondria, critical for treating numerous diseases, play a pivotal role in both human physiology and pathology. Several mitochondrial-targeting drugs are already commercially available, such as Idebenone, which crosses the blood–brain barrier and acts as a potent antioxidant to ameliorate cognitive dysfunction in chronic schizophrenia. Imeglimin, an oral antidiabetic medication, targets mitochondrial energy metabolism, affecting all three primary organs involved in glucose homeostasis: the liver, muscle and pancreas. Recent advancements in aging research have led to the development of targeted mitochondrial aging intervention supplements like Bioagen Pyrrovital Pro Enhanced Formula, drawing significant academic interest. Despite these advances, there are no commercially available mitochondria-targeted drugs for bone repair, although mitochondria are vital in bone cell metabolism and function. This gap indicates a promising direction for future research.

Pathological alterations in numerous prevalent diseases can arise from mitochondrial dysfunction. Given the similarity in mitochondrial damage patterns underlying various pathologies, the broad applicability of ‘mitochondrial therapy’ becomes evident. This review highlights and emphasizes the latest advances in mitochondrial-targeted biomaterial design for bone repair environments. The rapid increase in papers in this field over the past decade has demonstrated the vigorous development of mitochondrial-targeted materials. The rapid progress in material design principles has stimulated the potential of drugs or active ions with mitochondrial regulatory capabilities. Combining therapy and synergistic effects can be easily achieved by cleverly using these materials to treat diseases with complex biological mechanisms. As the world’s population ages, treating these prevalent diseases holds significant social, medical, and economic implications. However, the role of mitochondria as a biomaterial target remains to be considered in many aspects. The development of nanomaterials designed to target mitochondria is currently hampered by numerous challenges. These include the limited stability and water solubility of liposomes, suboptimal drug loading capacities in polymeric nanoparticles, and difficulties in effectively removing inorganic carriers. Furthermore, maintaining the stability and functional integrity of mitochondria is critical for the advancement of biomaterials targeting these organelles. This necessitates real-time monitoring of mitochondrial intermediate metabolite accumulation and the interaction with other organelles. Additionally, before translating these scientific advancements into clinical practice, a systematic evaluation of their feasibility, focusing on safety, efficacy and cost-effectiveness, is essential.

Our review article examines the critical role of mitochondria, essential subcellular organelles, in bone defect repair. Specifically, it emphasizes the significance of biomaterials that target mitochondrial functions, illustrating the direct correspondence between signaling pathways and mitochondrial activities. The article provides a comprehensive analysis of the mechanisms through which these materials influence bone healing. Research indicates that these biomaterials facilitate the differentiation of osteoblasts and enhance bone formation by modulating mitochondrial energy metabolism, the oxidative stress response and autophagy. Moreover, they indirectly improve the immune-inflammatory response during bone repair, mitigate the release of inflammatory mediators and encourage macrophage polarization towards healing phenotypes, thus expediting the repair of bone defects. So it follows that developing mitochondria-targeted biomaterials for bone defect repair remains a necessary and interesting research field. Through continuous research and innovation, we hope to develop more precise and efficient mitochondria-targeted multifunctional nanosystems. These systems can significantly improve treatment efficiency and accelerate the clinical translation of mitochondrial medicine, positively impacting patients’ recovery and quality of life.
